# Endoscopic Endonasal Approach of Congenital Meningoencephalocele Surgery: First Reported Case in Lithuania

**DOI:** 10.1155/2015/728561

**Published:** 2015-03-19

**Authors:** Svajūnas Balseris, Giedrius Strazdas, Saulius Ročka, Tomas Jakštas

**Affiliations:** ^1^Department of Ear, Nose and Throat Diseases, Republican Vilnius University Hospital, Siltnamiu 29, LT-04130 Vilnius, Lithuania; ^2^Department of Neurosurgery, Republican Vilnius University Hospital, Siltnamiu Gatvė 29, LT-04130 Vilnius, Lithuania; ^3^Department of Ear, Nose and Throat Diseases, LSMU Kauno Klinikos, Eiveniu Gatvė 2, LT-50009 Kaunas, Lithuania

## Abstract

Meningoencephalocele is a rare condition that usually occurs in children and is treated by neurosurgeons with occasional help from ENT doctors. The symptoms of meningoencephalocele might not develop until adulthood, but usually they are apparent immediately after birth. The case of small anterior basal transethmoidal meningoencephalocele in a 24-year-old patient who had headaches and runny nose since childhood is presented. Endonasal endoscopic approach for meningoencephalocele removal and skull base defect reconstruction was used. It was concluded that endoscopic endonasal approach is less invasive and provides an acceptable operative outcome with short recovery time and less postoperative complications in comparison with other external microsurgical approaches.

## 1. Introduction

Meningoencephaloceles (MECs) have been known since ancient times, but they were first described only in the 16th century [1]. The first monograph on MEC was written by Corvinus in 1749 [1], but as it is a rare condition and data was difficult to register and keep in old days, this condition is not very well known.

Two main forms are described: congenital MEC [2] and posttraumatic MEC [3]. Other forms, iatrogenic and spontaneous, are also described [4]. Meningoencephaloceles, based on the location, are classified into occipital MEC, MEC of the cranial vault, and frontoethmoidal (or sincipital) and basal MEC [1]. Frontoethmoidal (sincipital) and basal MEC are generally called anterior MEC.

According to Pianta et al., anterior MEC is more commonly found in Southeast Asia, Russia, and Central Africa regions [5]. It is a condition when the intracranial content protrudes through a defect in the skull that is located in the inferior part of anterior skull fossa, between lamina papyracea and processus clinoideus or in orbit [1]. Basal encephaloceles differ from sincipital ones, another subtype of anterior meningoceles, as they do not protrude externally [1]. Basal MECs are classified into transethmoidal, sphenoethmoidal, transsphenoidal, and frontosphenoidal [1].

Congenital MEC can also be present not as a single malformation of the skull but as a manifestation of a syndrome [1]. Basal MECs are occasionally linked to median cleft face and morning glory syndromes [6].

Cerebrospinal fluid (CSF) leakage, headaches, and nasal obstruction are the most common symptoms [6]. Relapsing meningitis and neurological symptoms might also be present, but it is highly unusual for all the symptoms to occur together [5]. Nasal endoscopy, biochemical nasal discharges test (if discharge is present), and both CT and MRI are important when diagnosing this pathology [5].

Because of the new technologies, endoscopic surgery innovations, and human desire to advance, functional endoscopic sinus surgery (FESS) was improved to a minimally invasive skull base anterior and middle fossa surgery during last decades and it is very important in basal meningoencephalocele treatment [7]. Endoscopic endonasal treatment approach with a two-layer skull base defect plasty was applied in this case.

## 2. Case Report

A 24-year-old patient was admitted to the Republican Vilnius University Hospital with the complaints of a constant runny nose and mild headache which has been bothering him since he was young. The patient was being treated from idiopathic runny nose and allergic rhinitis since he was a child. The patient, because of his constant headache, was treated by neurologists for intracranial hypertension syndrome that was diagnosed in 2005.

The patient presented runny nose, with a suspicion of a possible cerebrospinal fluid (CSF) leakage and a moderate headache during the examination. Nasal, nasopharyngeal, or pharyngeal obstruction symptoms were not present, Valsalva and Furstenberg tests were negative, and externally visible mass was not present. No changes were found during nasal endoscopy.

**Figure 1:**
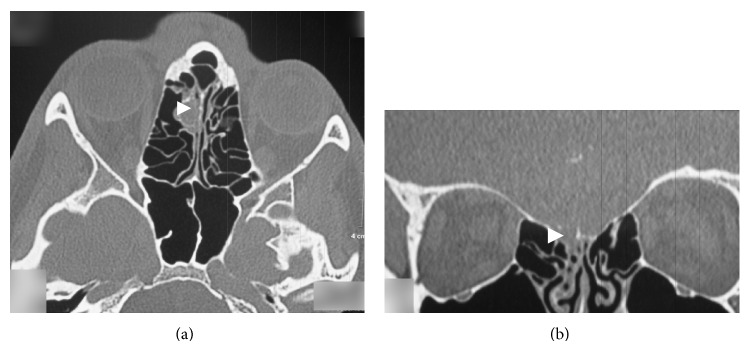
((a)-(b)) CT scan for patient with suspected CSF leakage. Defect in anterior ethmoidal cells in lamina cribrosa area.

**Figure 2:**
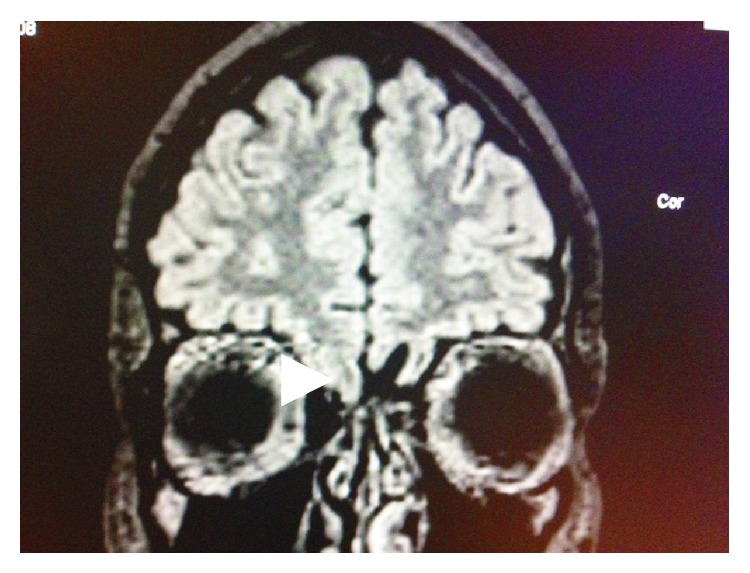
MRI for patient with skull base defect and possible CSF leakage. Gyrus rectus protrusion.

The patient complained about nasal discharge that was unilateral from the right side. 1 mL of nasal discharge in 10 minutes to a sterile test tube was collected. The fluid was clear and watery. It was examined and traces of protein were found and also 6.2 mmol/L of glucose level and cytosis of 1 cell in 3 visual fields; these results showed increased possibility of CSF leak. Defect in the skull base had to be found. CT scan was performed and a 1.8 cm × 0.5 cm sized bony defect in anterior ethmoidal cells in lamina cribrosa area (Figures 1(a) and 1(b)) was found. MRI was performed and gyrus rectus protrusion into ethmoidal cells was noticed (Figure 2). Transethmoidal meningoencephalocele and right sided nasal liquorrhea diagnosis was made.

Endonasal endoscopic approach with double layer surgical technique for defect closure was chosen as treatment. Surgery consisted of two steps: first step was to remove the MEC and the second was a skull base defect plasty.

**Figure 3:**
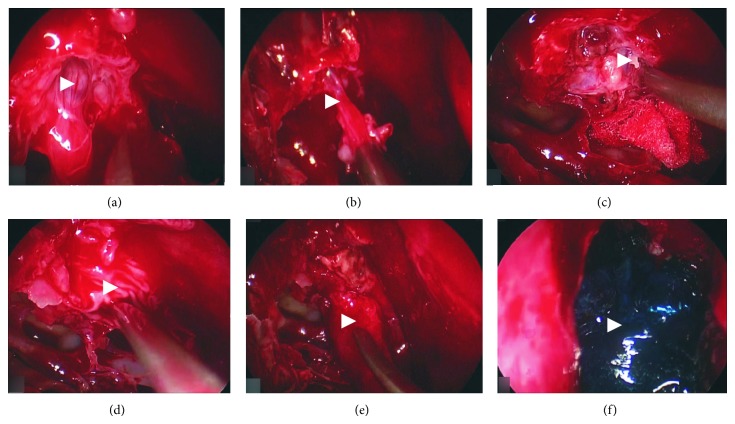
Steps of operation. (a) Bony defect and CSF leakage are identified. (b) Location of meningoencephalocele is established. (c) Sharp fragments of bony defect are being removed. (d) Temporal fascia flap is inserted between dura mater and bone. (e) Middle turbinate flap is covering the outer part of the defect. (f) Nasal cavity filled with synthetic glue (Duraseal).

Maxillary sinus, frontal sinus, and frontal recess were opened. Anterior and posterior ethmoidal cells were removed and bony defect and CSF leakage were identified (Figure 3(a)). Location of meningoencephalocele was ascertained (Figure 3(b)). Temporal fascia was taken as first, intracranial flap and mucosa-periosteum layer from the middle turbinate was chosen as second, extracranial flap, for the bony defect correction.

Lumbar drainage was performed during the surgery to lower the intracranial pressure. Sharp fragments of the bony defect were removed and the surface was prepared for the correction (Figure 3(c)). The stalk of MEC was cauterized and MEC sack was removed. Dura mater was separated circularly 2-3 millimeters from the bone; then, mucosa was circularly separated from the bony defect from nasal side. Temporal fascia flap was inserted between dura mater and the bone intracranially (Figure 3(d)). The middle turbinate flap was used to cover the outer part of the defect (Figure 3(e)). Synthetic glue and nasal tamponade were used during the operation (Figure 3(f)).

Nasal tamponade was removed two days after surgery; lumbar drainage was stopped 5 more days later. The patient recovered and was discharged after 8 days in total without any previously shown pathological symptoms.

**Figure 4:**
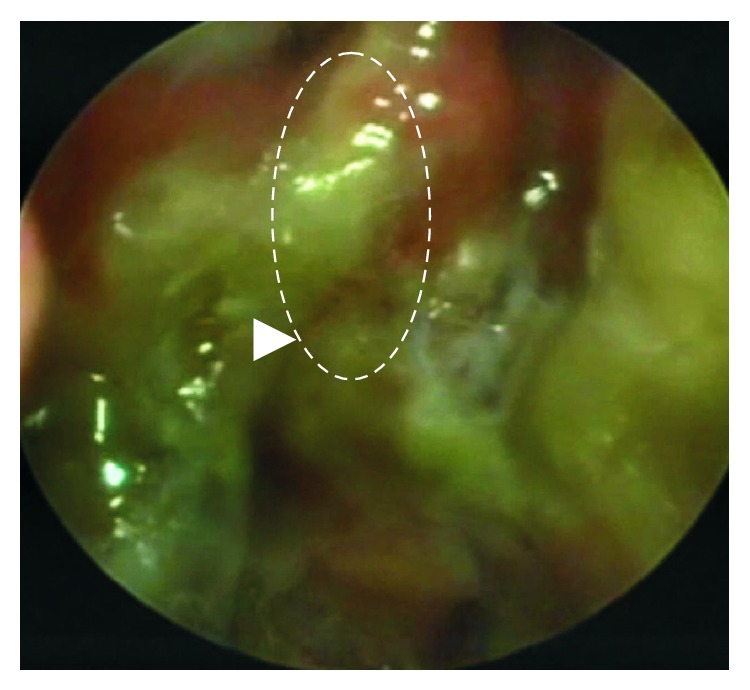
Nasal endoscopy after 3 weeks. Postoperative nasal scaring and traces of Duraseal.

**Figure 5:**
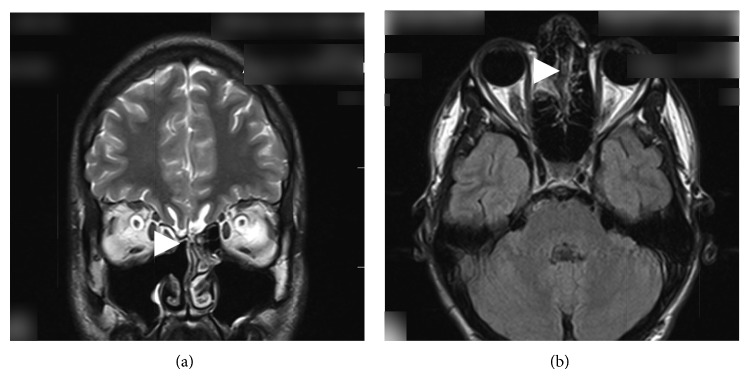
((a)-(b)) Control MRI after 8 months. The defect in the skull base is sealed; there is no more CSF leakage and no brain protrusion.

Endoscopic examination was performed 3 weeks later: traces of glue were visible, maxillary and frontal sinuses were clear, and the defect was sealed (Figure 4). MRI was performed after 8 months showing only postoperative intranasal scaring and excellent recovery results with no previously shown symptoms and no CSF leakage (Figures 5(a) and 5(b), after operation).

## 3. Discussion

Meningoencephalocele is a rare condition; its incidence varies from 0.008% to 0.03% in different parts of the world, whereas prevalence of basal MEC is 1 in 35,000 live births [5, 8]. In this case, patient had rare anterior basal transethmoidal localization and it was the first case in Republican Vilnius University Hospital in 20 years. Also, it was the first reported case across the country.

According to Tirumandas et al. [6], basal transethmoidal MECs are usually present together with nasal obstruction and as the mass is visible with nasal endoscopy, CT, or MRI, clinical diagnosis is made in early childhood. In this case, the MEC was small and was not visible during nasal endoscopy due to its localization in anterior ethmoidal cells.

MEC based on the development can be classified into congenital, acquired, or idiopathic/spontaneous. Only posttraumatic or infectious acquired MEC is easy to distinguish from others. Though a defect in the skull base can be a congenital malformation and MEC protrudes through it later in life as a result of various causes [1, 8], it was suspected that our patient had a congenital skull base malformation as he did not have any head traumas, surgical operations, or infections and the symptoms were not obvious during his life. It was suspected that symptoms started to develop because of his hydrocephalus. Increased pressure in cranium and a present skull base defect caused part of the brain and meninges to prolapse. Constantly increased CSF pressure caused headaches and constant CSF leak.

Congenital MECs are often linked to various syndromes, posterior MECs are usually linked to Meckel, Pseudo-Meckel, and Chemke syndromes, and anterior MECs are usually present in frontonasal dysplasia and amniotic band syndrome (both for sincipital) [1]. Basal MECs are less common [9] and so are the syndromes that they can be linked to. Basal MECs are occasionally linked to median cleft face and morning glory syndromes [6]. But these possible syndromes were not visible in our patient, so it was assumed that his condition is a single malformation.

Signs and symptoms of MEC vary. It can be visible as a mass protruding externally, visible on nasal endoscopy or not visible at all, depending on the localization. Runny nose complaints are quite common; nasal, nasopharyngeal, or pharyngeal obstruction symptoms can also be present [1, 5]. Relapsing meningitis and sinusitis also can occur [5, 6], as MEC opens a direct route for ascending bacteria to travel and induce inflammation in superior structures. In addition, positive Furstenberg or Valsalva tests might be present [6, 10]. Only two symptoms, constant runny nose and headache, were present in our patient; idiopathic runny nose was treated by allergist and headache by neurologists. Patients who have only one symptom are also described in literature (Habu et al. [11]). Both Furstenberg and Valsalva tests were negative in our case.

Nasal endoscopy is a valuable test for visualizing mass in the nasal cavity and it is helpful for differential diagnosis [5]. As the mass was not visible during nasal endoscopy, MEC could not be excluded as a possible diagnosis as CSF leakage was suspected because of constant nasal discharge. It was collected and examined for protein, glucose, and cytosis. This test is quite unspecific, though it also raises a possibility of CSF leakage and is cheaper than more specific tests such as fluorescein or beta-transferrin tests that are used widely nowadays [5, 12]. The bony defect in the skull base can be discerned only by CT or MRI scan and it enables soft tissues and content of the suspected mass assessment [6]. After CT and MRI, the bony defect of the skull base was discerned and the protrusion of the brain through the defect was assessed.

Treatment of anterior basal MEC is still under development. Functional endoscopic sinus surgery (FESS) was introduced in the early 1980s; Ear, nose, and throat (ENT) surgeons have pioneered the use of the endoscopic technique to diagnose and to treat chronic inflammatory and benign lesions of the nasal and paranasal sinuses [7]. The first FESS in Lithuania was performed at the Republican Vilnius University Hospital by doctor J. L. Martinkėnas in 1992 [13]. For the management of CSF leaks, paranasal sinuses neoplasms, and meningoencephaloceles, surgeons Carrau, Hao, and Locatelli and others started to use the same route after ten years [7, 14, 15]. The first time in Lithuania that iatrogenic CSF leak using endonasal endoscopic approach was managed was in 1997; the first sinus neoplasms were removed in 2005, skull base plasty was started in 2009 [13], and the first endonasal endoscopic approach for transethmoidal MEC was used in 2013.

This experience improved the knowledge of the anatomy of this area and resulted in the evolution of the endoscopic approach to the anterior skull base [1]. This method still encounters problems as it is still being developed for routine use. Various theoretical, practical, and research works have to be done before these skills will be mastered. The endoscopic endonasal technique for the management of lesions of sphenoethmoidal region became more popular for CSF leaks, meningocele, and MEC, because of lower surgical morbidity compared to the other techniques [1, 14, 15]. Endoscopic approach started to emerge to medical world as the treatment of choice for these patients, instead of neurosurgical frontal craniotomy that was pushed aside. Endoscopic approach was chosen for the first time in the Republican Vilnius University Hospital in our case as it is less traumatizing and the experience of surgeons with endoscopic procedures is quite high. The technique of skull base defect correction is the other area of development. According to literature (Pianta et al. [5]), there are different surgical techniques: one-layer, two-layer, underlay, overlay, or tobacco pouch [5]. Anterior transethmoidal meningoceles are usually operated on using double layer technique with fascia for duraplasty, though various other materials like temporal muscles, septal mucoperichondrium, turbinate bone, and others can be used [5]. The second layer might vary: middle turbinate flap, nasoseptal flap, or others [5]. Most of the used flaps are equally good and the choice for duraplasty and bony defect closure only depends on the surgeon [5]. Two-layer technique was applied in the presented case; the temporal fascia was chosen for the first layer and the mucosa-periosteum flap from the middle turbinate for the second layer. Fibrin or synthetic glue can be used for skull base defect correction; in this case, synthetic glue was applied after two layers of grafts.

New technologies, endoscopic surgical innovations, and human desire to advance [16] during the last decades enabled FESS to be expanded to minimally invasive skull base anterior and middle fossa surgery, which is suitable for basal meningoencephalocele and skull base plasty. We believe that endoscopic endonasal approach allows less invasive surgery and provides an acceptable outcome in comparison with other approaches.

## 4. Conclusion

Anterior basal MEC is a condition that rarely occurs in clinical practice. Though if possible anterior basal MEC and possible nasal liquorrhea are suspected by a specialist during the examination, patient history and complaints need to be considered before performing nasal endoscopy, CT, or MRI. Nowadays, developing technologies and the experience of ENT doctors enable operating using endonasal endoscopic approach. It becomes a treatment of choice for these patients instead of neurosurgical frontal craniotomy.

## References

[1] Jimenez D. F., Barone C. M., Albright A. L., Adelson P. D., Pollack I. F. (2008). Encephaloceles, meningoceles, and dermal sinuses. *Principles and Practice of Pediatric Neurosurgery*.

[2] Garg P., Rathi V., Bhargava S. K., Aggarwal A. (2005). CSF rhinorrhea and recurrent meningitis caused by transethmoidal meningoencephaloceles. *Indian Pediatrics*.

[3] Norris E. B., Cooper P., Juurlink D. N. (2006). A case of recurrent meningitis. *Canadian Medical Association Journal*.

[4] Samadian M., Moghaddasi H., Vazirnezami M. (2012). Transcranial approach for spontaneous csf rhinorrhea due to Sternberg's canal intrasphenoidal meningoencephalocele: case report and review of the literature. *Turkish Neurosurgery*.

[5] Pianta L., Pinelli L., Nicolai P., Maroldi R., Maroldi R., Nicolai P. (2005). Cerebrospinal fluid leak, meningocele and meningoencephalocele. *Imaging in Treatment Planning for Sinonasal Diseases*.

[6] Tirumandas M., Sharma A., Gbenimacho I. (2013). Nasal encephaloceles: a review of etiology, pathophysiology, clinical presentations, diagnosis, treatment, and complications. *Child's Nervous System*.

[7] De Notaris M., Esposito I., Cavallo L. M. (2008). Endoscopic endonasal approach to the ethmoidal planum: Anatomic study. *Neurosurgical Review*.

[8] Suwanwela C. (1972). Geographical distribution of fronto-ethmoidal encephalomeningocele. *British Journal of Preventive & Social Medicine*.

[9] Kubo A., Sakata K., Maegawa J., Yamamoto I. (2005). Transethmoidal meningoencephalocele in an elderly woman. Case report. *Neurologia Medico-Chirurgica (Tokyo)*.

[10] Abdel-Aziz M., El-Bosraty H., Qotb M. (2010). Nasal encephalocele: endoscopic excision with anesthetic consideration. *International Journal of Pediatric Otorhinolaryngology*.

[11] Habu M., Niiro M., Toyoshima M., Kawano Y., Matsune S., Arita K. (2009). Transethmoidal meningoencephalocele involving the olfactory bulb with enlarged foramina of the lamina cribrosa—case report. *Neurologia Medico-Chirurgica*.

[12] Middeiweerd M. J., de Vries N., Calliauw J., van Kamp G. J. (1995). A new biochemical assay in the diagnostic management of nasal cerebrospinal fluid leakage. *European Archives of Oto-Rhino-Laryngology*.

[13] Martinkėnas J. L., Martinkėnas J. L., Virgilijus S. (2012). Sinusų endoskopinės chirurgijos principai. *Rinologijos pradmenys*.

[14] Hao S. P., Wang H. S., Lui T. N. (1995). Transnasal endoscopic management of basal encephalocele—craniotomy is no longer mandatory. *The American Journal of Otolaryngology—Head and Neck Medicine and Surgery*.

[15] Locatelli D., Rampa F., Acchiardi I., Bignami M., de Bernardi F., Castelnuovo P. (2006). Endoscopic endonasal approaches for repair of cerebrospinal fluid leaks: nine-year experience. *Neurosurgery*.

[16] Stammberger H., Posawetz W. (1990). Functional endoscopic sinus surgery. Concept, indications and results of the Messerklinger technique. *European Archives of Oto-Rhino-Laryngology*.

